# CO_2_-facilitated upcycling of polyolefin plastics to aromatics at low temperature

**DOI:** 10.1093/nsr/nwae097

**Published:** 2024-03-15

**Authors:** Yi Ding, Shuchi Zhang, Cheng Liu, Yu Shao, Xiulian Pan, Xinhe Bao

**Affiliations:** State Key Laboratory of Catalysis, Dalian Institute of Chemical Physics, Chinese Academy of Sciences, Dalian 116023, China; State Key Laboratory of Catalysis, Dalian Institute of Chemical Physics, Chinese Academy of Sciences, Dalian 116023, China; University of Chinese Academy of Sciences, Beijing 100049, China; State Key Laboratory of Catalysis, Dalian Institute of Chemical Physics, Chinese Academy of Sciences, Dalian 116023, China; University of Chinese Academy of Sciences, Beijing 100049, China; State Key Laboratory of Catalysis, Dalian Institute of Chemical Physics, Chinese Academy of Sciences, Dalian 116023, China; Department of Chemical Physics, University of Science and Technology of China, Hefei 230026, China; State Key Laboratory of Catalysis, Dalian Institute of Chemical Physics, Chinese Academy of Sciences, Dalian 116023, China; State Key Laboratory of Catalysis, Dalian Institute of Chemical Physics, Chinese Academy of Sciences, Dalian 116023, China; Department of Chemical Physics, University of Science and Technology of China, Hefei 230026, China

**Keywords:** polyolefins, carbon dioxide, aromatics, upcycling, bifunctional catalysis

## Abstract

Plastics are one of the most produced synthetic materials and largest commodities, used in numerous sectors of human life. To upcycle waste plastics into value-added chemicals is a global challenge. Despite significant progress in pyrolysis and hydrocracking, which mainly leads to the formation of pyrolysis oil, catalytic upcycling to value-added aromatics, including benzene, toluene and xylene (BTX), in one step, is still limited by high reaction temperatures (>500°C) and a low yield. We report herein CO_2_-facilitated upcycling of polyolefins and their plastic products to aromatics below 300°C, enabled by a bifunctional Pt/MnO_x_-ZSM-5 catalyst. ZSM-5 catalyzes cracking of polyolefins and aromatization, generating hydrogen at the same time, while Pt/MnO_x_ catalyzes the reaction of hydrogen with CO_2_, consequently driving the reaction towards aromatization. Isotope experiments reveal that 0.2 kg CO_2_ is consumed per 1.0 kg polyethylene and 90% of the consumed CO_2_ is incorporated into the aromatic products. Furthermore, this new process yields 0.63 kg aromatics (BTX accounting for 60%), comparing favorably with the conventional pyrolysis or hydrocracking processes, which produce only 0.33 kg aromatics. In this way, both plastic waste and the greenhouse gas CO_2_ are turned into carbon resources, providing a new strategy for combined waste plastics upcycling and carbon dioxide utilization.

## Teaser

CO_2_ facilitates upcycling of polyolefins to aromatics below 300 °C in one step, with 90% CO_2_ being incorporated into the products, which provides a new strategy for combined waste plastics upcycling and carbon utilization.

## INTRODUCTION

Plastics are widely used in various fields, such as packaging, agriculture, construction and automobiles, making our lives convenient and facilitating industrial processes [1,2]. The global production of plastics was reported to reach 9.2 billion tons in 2020 [3], and it is still increasing every year. Among them, polyolefins, represented by polyethylene (PE) and polypropylene (PP), account for ∼55% of all plastics [4,5]. However, only ∼10% of plastic waste is recycled due to chemical inertness, while 70% is burned and landfilled as garbage, and the remaining 20% is just discarded in nature [6,7]. This has caused severe environmental problems [1,3,4]. It is a global challenge to upcycle plastics, for instance turning them into value-added chemicals such as aromatics, particularly benzene, toluene and xylene (BTX), to keep their carbon footprint as small as possible. BTX are widely used as feedstocks for synthesis of degradable plastic, rubber, dyes, pharmaceuticals, etc. [8,9].

However, the C−C bond of polyolefin skeletons is rather stable, with a bond energy of 348 kJ/mol [10]. Conventional technologies for upcycling polyolefins involve high-temperature pyrolysis or hydrocracking. The C−C bonds cleave randomly and irregularly, resulting in a wide distribution of products such as pyrolysis oil, which exhibit a broad boiling range [11–14]. Recently, Lercher and co-workers reported an elegant upcycling strategy by combining endothermic polyolefin cracking with exothermic alkylation reactions using chloroaluminate ionic liquids as catalysts, which selectively converted PE and PP to C_6_−C_10_ iso-paraffins [15]. Zhang *et al.* [16] reported an interesting work on PE conversion to aromatics using a Pt/γ-Al_2_O_3_ catalyst at 280°C. The main products were long-chain alkyl-aromatics. The selectivity for monocyclic aromatics was 22%, which was accompanied by 48% alkanes and cycloalkanes. There was almost no BTX produced. To achieve a higher yield of BTX, reaction temperatures as high as 500–600°C and two reactor stages were commonly employed, with pyrolysis in one reactor followed by catalytic aromatization in the second reactor, similar to fluid catalytic cracking and catalytic reforming in the petrochemical industry [17–19]. At the same time, extensive efforts were made to explore one-pot catalytic conversion to aromatics [20]. For instance, Garforth *et al.* studied the catalytic conversion of polyolefins to aromatics in fluidized bed reactors using zeolites as catalysts, and HZSM-5 was demonstrated to give the highest BTX yield of 5.1% at 430°C [21]. Du *et al.* [22] recently reported that high-density PE was converted into aromatics with a yield of 44.5%, and BTX accounted for ∼8% of aromatics, using Ru/HZSM-5 as a catalyst at 280°C.

In our search for a more selective approach for valorization of polyolefin plastics, we looked into the thermodynamics of polyolefin conversion using C_20_-olefin to represent the feed and toluene as a representative desired aromatic product (Equations S1 and S2). The thermodynamics favor the generation of alkanes via hydrogen transfer reaction, and thus the yield of aromatics is limited. For instance, formation of 1 mol toluene is accompanied by 3 moles alkanes (represented by propane). We propose herein to upcycle polyolefins with CO_2_ as a hydrogen sink (Equation S3). By introducing CO_2_ into the reaction system, the reaction is expected to be driven towards aromatics. It does not only suppress alkane formation but also enhances the aromatization, i.e. aromatics yield could increase almost to two times in comparison to the reaction without CO_2_ at thermodynamic equilibrium at 300°C (Fig. S1). Just recently, Zhang *et al.* [23] reported the upcycling of PE with CO_2_; the yield of aromatics increased from 30.7 wt% to 40.4 wt% while CO_2_ was reduced to CO over a catalyst composed of Cu-Fe_3_O_4_ and Zn/ZSM-5 at 390°C. If CO_2_ is reduced to hydrocarbons and H_2_O, the process would have benefited additionally from its exothermicity in combination with endothermic polyolefin aromatization, which could allow a reaction at milder conditions. To do so, we started with an oxide-zeolite (OXZEO) catalyst for CO hydrogenation to aromatics [24–26], i.e. MnO_x_-ZSM-5. To facilitate the hydrogenation of CO_2_, we introduced Pt onto MnO_x_. Thus, a bifunctional Pt/MnO_x_-ZSM-5 catalyst is designed. The results validate well the catalyst design concept, as it efficiently catalyzes conversion of different types of polyolefins, including PE, PP, plastic PE film and PP bottles, into aromatics below 300°C. Taking low-density PE with a weight average molecular weight of *M_w_* = 2.9 × 10^5^ g·mol^−1^ (denoted as LDPE-1) as an example, the yield of aromatics was as high as 64% at 300°C, and monocyclic aromatics account for 87% and BTX for 60% of the yield. These yields are twice as high as those achieved in catalytic cracking and hydrocracking over the same catalyst. More importantly, the new process approaches CO_2_ neutrality with the upcycling of plastics, as 90% of consumed CO_2_ was incorporated into the aromatic products.

## RESULTS AND DISCUSSION

### Polyolefin upcycling reaction

**Figure 1.**
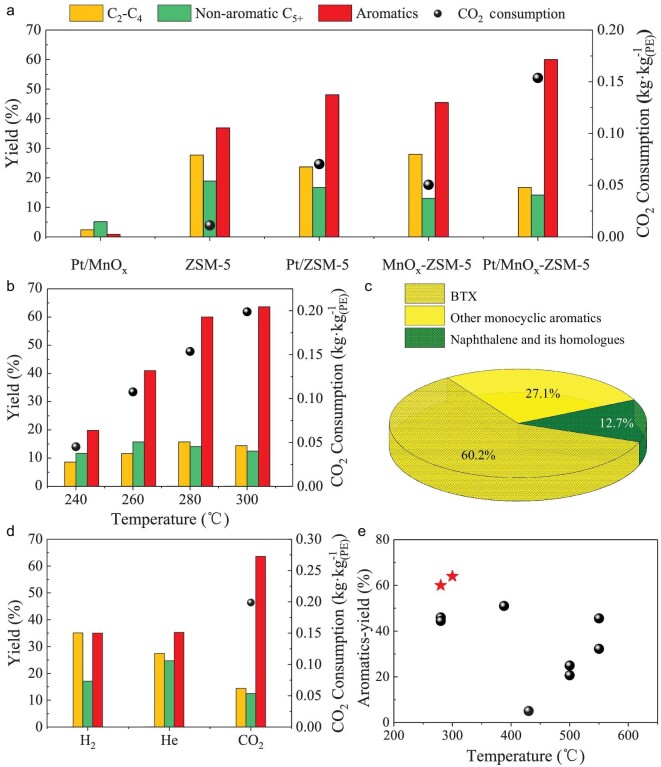
Catalytic performance of Pt/MnO_x_-ZSM-5 in the upcycling of LDPE-1 with CO_2_. (a) Performance in comparison to Pt/MnO_x_, ZSM-5, Pt/ZSM-5 and MnO_x_/ZSM-5 catalysts. Reaction conditions: 1.0 MPa CO_2_, 280°C, 0.4 g catalyst, 1.0 g LDPE-1, 100 mL batch reactor. (b) Performance as a function of reaction temperature. (c) Distribution of aromatic products. (d) Product yields in the presence of H_2_, He and CO_2_ at 300°C. (e) Aromatics yield in this work (denoted by red stars) in comparison to the previously reported values (black circles) for one-pot reactions [16,20–22,27–29].

X-ray diffraction (XRD) only detected the MnO crystal phase in the H_2_-reduced Pt/MnO_x_ (Fig. S2) and high-resolution transmission electron microscopy (HRTEM) results revealed homogeneously dispersed Pt nanoparticles with an average size of ∼2.0 nm (Fig. S3). The reaction conditions were optimized, including reaction time (Fig. S5), CO_2_ pressure (Fig. S6), Pt loading (Fig. S7), mass ratio of Pt/MnO_x_ to ZSM-5 (Fig. S8) and charges of polyolefin and catalyst (Fig. S9), as well as the mass ratio of polyolefin to catalyst (Fig. S10) and the reactor volume (Fig. S11). Then all reaction tests were carried out under 1.0 MPa CO_2_ in a 100 mL batch reactor (Scheme S1), using 1.0 wt% Pt/MnO_x_ with a Pt/MnO_x_ : ZSM-5 mass ratio of 1 : 1 unless otherwise stated. LDPE-1 (Table S2) was almost completely converted after 3 h at 280°C (Fig. S5). The sum yield of all hydrocarbon products reached 90% (Table S3). Figure 1b shows that the aromatics yield reached 64% (Table S4). At the same time, CO_2_ consumption was 0.2 kg·kg_(PE)_^−1^. The monocyclic aromatics account for 87% and BTX for 60% among the aromatics (Fig. 1c and Fig. S13). There were only 25% alkanes with almost no CH_4_ and little coke (Table S4).

By contrast, Pt/MnO_x_ alone exhibited very weak activity, giving only a small amount of pyrolysis oil (yield 5%) and C_2_−C_4_ hydrocarbons (yield 2%), with few aromatics detected (Fig. 1a). Although ZSM-5 zeolite alone could catalyze the cracking and aromatization of polyolefins, there was little CO_2_ consumption, and the aromatics yield (37%) was only about half of that over the bifunctional catalyst Pt/MnO_x_-ZSM-5, while the yield of alkanes was 50% (Table S3). MnO_x_-ZSM-5 was also effective in converting LDPE-1 to aromatics. Its yield was still lower (44%), and the consumption of CO_2_ was only one-third of that over Pt/MnO_x_-ZSM-5. The above results demonstrate clearly the essential benefits of bifunctionality provided by the combination of Pt/MnO_x_ and ZSM-5.

Replacing CO_2_ with H_2_ or He in the reaction (Fig. 1d) clearly demonstrates the beneficial role of CO_2_ in the upcycling of PE to aromatics over Pt/MnO_x_-ZSM-5. For instance, the reaction in H_2_, i.e. mimicking hydrocracking, gave only half the aromatics yield (35%) and was further accompanied by alkanes with a yield (51%) of two times higher than that in the reaction with CO_2_ (Table S4). Similarly, the reaction of LDPE-1 in He also gave a low aromatics yield (35%) and high alkanes yield (44%). Table S4 further shows that the presence of CO_2_ reduces coke formation in comparison to the reactions in the presence of He and H_2_.

These data clearly demonstrate that CO_2_ suppresses alkane formation by scavenging hydrogen, which significantly boosts the aromatization of olefinic intermediates. Figure 1e and Fig. S14 (corresponding to Table S5) reveal that this new CO_2_-facilitated upcycling process gives a distinctly higher aromatics yield and BTX yield in comparison to previously reported processes for one-pot upcycling of polyolefins [16,20–22,27–29], even though the reaction temperature is lower than 300°C.

**Figure 2.**
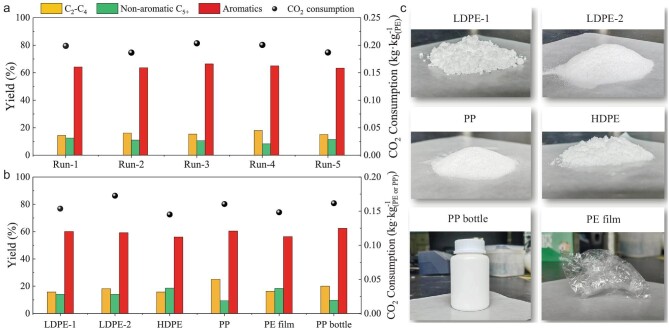
Catalytic performance of Pt/MnO_x_-ZSM-5. (a) Recyclability test in the upcycling of LDPE-1 with CO_2_ at 300°C. (b) Activity in the catalytic conversion of various polyolefins and plastic products at 280°C. (c) Polyolefins and plastic products used in Fig. 2b. Reaction conditions: 1.0 MPa CO_2_, 0.4 g catalyst, 1.0 g polyolefins, 100 mL batch reactor.

The recyclability of the catalyst was studied by calcining the used Pt/MnO_x_-ZSM-5 catalyst in air at 600°C, followed by reduction at 320°C. Figure 2a shows practically no loss of activity even after four such straightforward and rather practicable regeneration cycles. Thus, no irreversible deactivation was observed, indicating a rather robust catalyst. The particle size of Pt and MnO_x_, and ZSM-5 crystallinity, did not change much and the acidic sites were recovered after calcination and reduction when compared to the fresh Pt/MnO_x_-ZSM-5 catalyst (Figs S15–S18). Further experiments revealed the versatility of the bifunctional Pt/MnO_x_-ZSM-5 catalyst for the upcycling of different types of polyolefins, including LDPE-2 with a weight average molecular weight (*M_w_* = 4.8 × 10^5^ g·mol^−1^), high density PE (HDPE, *M_w_* = 2.5 × 10^5^ g·mol^−1^), PP (*M_w_* = 4.7 × 10^4^ g·mol^−1^), and plastic products such as kitchen PE film (*M_w_* = 3.5 × 10^5^ g·mol^−1^) and PP bottles (*M_w_* = 6.8 × 10^6^ g·mol^−1^). The data in Fig. 2b demonstrate that the various polyolefins were all efficiently converted and the aromatics yield ranged from 56% to 61% at 280°C.

### Reaction mechanism

**Figure 3.**
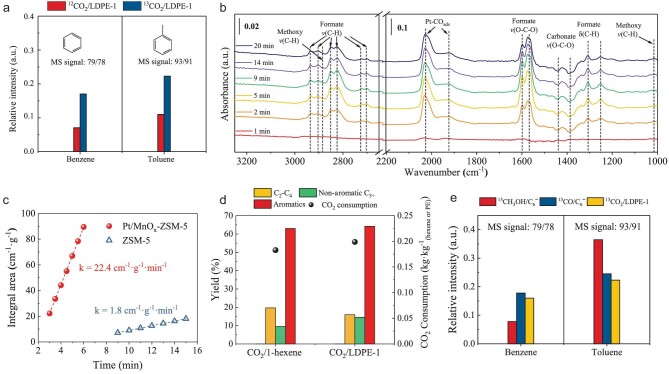
Mechanistic understanding of the CO_2_-facilitated upcycling of polyolefins over Pt/MnO_x_-ZSM-5. (a) The relative intensity ratio of mass spectrum signals m/z = 79/78 (^13^CC_5_H_6_/C_6_H_6_) and m/z = 93/91 (^13^CC_6_H_8_/C_7_H_8_), corresponding to ^13^CC_5_H_6_/C_6_H_6_ (benzene) and ^13^CC_6_H_8_/C_7_H_8_ (toluene) in a control reaction of ^13^CO_2_ with LDPE-1 (^13^CO_2_/LDPE-1), in comparison to that of ^12^CO_2_/LDPE-1 at 300°C. (b) *In situ* IR differential spectra recorded upon exposing the CO_2_-adsorbed Pt/MnO_x_ (pre-reduced) to H_2_ (5 mL/min). (c) Formation rates of Si-OD-Al measured for Pt/MnO_x_-ZSM-5 (filled dots) and ZSM-5 (open squres) during H-D exchange experiments. (d) A control reaction of CO_2_ with 1-hexene (CO_2_/C_6_^=^) in comparison to that of CO_2_/LDPE-1 at 300°C. (e) The relative intensity ratio of m/z = 79/78 and m/z = 93/91 in the reaction of ^13^CO_2_/LDPE-1 in comparison to control reactions of ^13^CH_3_OH/C_6_^=^ and ^13^CO/C_6_^=^.

To elucidate the role of CO_2_, we used ^13^CO_2_ as a feedstock for conversion of LDPE-1. Figure 3a displays the m/z = 79/78 and m/z = 93/91 signals in the products, which correspond to ^13^CC_5_H_6_/C_6_H_6_ (benzene) and ^13^CC_6_H_8_/C_7_H_8_ (toluene), respectively. They were significantly higher than the corresponding natural ^13^C abundance ratios obtained in the reaction of ^12^CO_2_/LDPE-1 (represented by the red bar). This clearly demonstrates the incorporation of ^13^C into the benzene ring. In comparison, no ^13^C was incorporated into detected alkanes (Fig. S19). Furthermore, ^13^CO was detected in the products (Fig. S20) due to reactions S4–S5. According to the relative concentration of ^13^CO among all CO (86%) and the amount of the converted CO_2_, one can calculate that 90% of the consumed ^13^CO_2_ had been incorporated into aromatics and the rest was released as ^13^CO according to Equation S6.

Since CO_2_ can be activated over Pt/MnO_x_ in the composite catalyst, we turned to *in situ* Fourier transform infrared (FTIR) spectroscopy to monitor CO_2_ adsorption and its hydrogenation for further understanding. As displayed in Fig. S21, bridged carbonate and carboxylate species showed up on reduced Pt/MnO_x_ upon CO_2_ adsorption, as well as linearly adsorbed CO (2045 and 1970 cm^−1^) over the Pt and Pt/MnO_x_ interface [30–32]. Upon subsequent introduction of H_2_ (Fig. 3b), carbonate (1401 cm^−1^) was transformed into bidentate and monodentate formate (1530–1620 cm^−1^) [33–35] and methoxy (2908 and 1039 cm^−1^) [36–39] species. At the same time, the linearly adsorbed CO band intensified, validating the reduction of CO_2_. By contrast, only carbonate species were observed over MnO_x_ alone and subsequent H_2_ introduction only led to slightly weakened bands (Fig. S22). These results demonstrate the essential role of Pt/MnO_x_ for reduction of CO_2_, leading to formation of CH_x_O and CO.

CO_2_ can be reduced by hydrogen, which is generated during aromatization of olefins. H-D exchange experiments over Pt/MnO_x_-ZSM-5 (Fig. S23) show that the Si-OD-Al FTIR band [40,41] (2658 cm^−1^) readily formed and rapidly intensified with time, compared to the corresponding band over pure ZSM-5 that remained rather weak even after an extended time. The Si-OD-Al formation rate (22.4 cm^−1^ g^−1^ min^−1^) was estimated to be more than one order of magnitude higher than that over ZSM-5 (1.8 cm^−1^ g^−1^ min^−1^), according to the integral area of the infrared bands (Fig. 3c and Fig. S23). The facilitated H-D exchange clearly evidences deuterium dissociation and spillover on Pt/MnO_x_ and subsequent transport to ZSM-5. Hydrogen spillover between physically mixed phases is frequently observed [42–44]. Thus, hydrogen spillover could facilitate hydrogen capture by CO_2_ over Pt/MnO_x_.

To simplify the reaction and capture the primary product of polyolefin cracking in CO_2_, we packed the physically mixed ZSM-5 and LDPE-1 as a fixed bed and CO_2_ was fed through (Scheme S2). The results in Fig. S24 demonstrate that LDPE-1 was mainly cracked to C_3_−C_7_ olefins with a yield of over 70%. Therefore, we took 1-hexene as a representative olefin for further model reactions in the batch reactor. Figure 3d shows that the reaction of CO_2_ with1-hexene gave a similar product distribution and similar aromatics yield of 63% over Pt/MnO_x_-ZSM-5 as those in the reaction of CO_2_ with LDPE-1. Therefore, the reaction of CO_2_ with PE likely proceeds via initial cracking of PE into C_3_−C_7_ olefins followed by their aromatization. This was further verified by control reactions of H_2_/1-hexene, He/1-hexene and CO_2_/1-hexene, for comparison to those with LDPE-1 (Fig. S25).

To understand whether CO_2_ is incorporated into the products via CO- or methanol-derived reaction intermediates, control reactions of 1-hexene (C_6_^=^) with ^13^CH_3_OH and ^13^CO, respectively, were carried out over Pt/MnO_x_-ZSM-5. Both reactions led to formation of aromatics. The data in Fig. 3e show that the ratio of the m/z = 79/78 signal (corresponding to ^13^CC_5_H_6_/C_6_H_6_) and that of the m/z = 93/91 (^13^CC_6_H_8_/C_7_H_8_) from the ^13^CO/C_6_^=^ feed are similar to those observed for ^13^CO_2_/LDPE-1 reaction, whereas the 79/78 ratio is ca. 50% lower and the 93/91 ratio is ca. 33% higher in the case of ^13^CH_3_OH/C_6_^=^ feed. This strongly suggests that CO_2_ incorporation into BTX proceeds via CO rather than CH_3_OH-derived intermediates.

Further control reactions of CO/C_6_^=^ gave a similar aromatic distribution to the reaction of CO_2_/LDPE-1, but that of CH_3_OH/C_6_^=^ differed under the same conditions (Fig. S26). This further suggests that the reaction unlikely occurs via CH_3_OH-derived intermediates. Another control reaction, i.e. CO/LDPE-1, gave a similar product distribution and aromatics-yield as those in the reaction of CO_2_/LDPE-1 (Fig. S27). These results further support CO or CO derivatives as intermediates over Pt/MnO_x_-ZSM-5. It is worth noting that the produced CO is not converted to alkanes, as evidenced by almost no ^13^C containing alkanes in a control reaction of ^13^CO/1-hexene (Fig. S28).

**Scheme 1.**
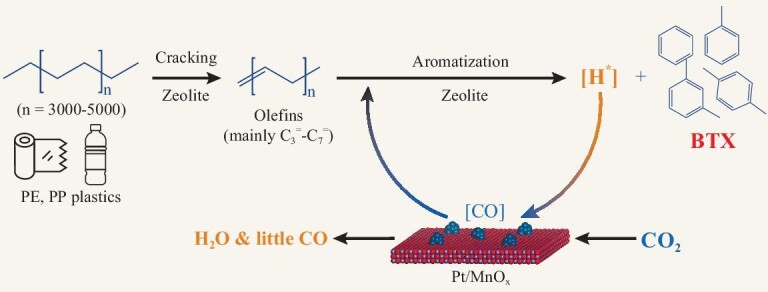
Reaction pathway proposed for the CO_2_-facilitated upcycling of polyolefins to aromatics (BTX), enabled by a bifunctional Pt/MnO_x_-ZSM-5 catalyst.

Thus, we propose the following reaction pathway for CO_2_-facilitated upcycling of polyolefins. As depicted in Scheme 1, polyolefins first crack, forming mainly C_3_−C_7_ olefins over ZSM-5. Subsequently, they are aromatized, generating hydrogen at the same time. Hydrogen species are scavenged by CO_2_ over Pt/MnO_x_, forming CO and H_2_O. Hence, hydrogenation of olefinic reaction intermediates to alkanes is suppressed, leading to an enhanced aromatics yield. Furthermore, the resultant CO and its derived intermediates are incorporated into aromatics, similar to CO hydrogenation to aromatics in the OXZEO catalysis [24,45], which is an additional benefit. The stoichiometric analysis shows that 25.2 mol C (carbon-atom-based) of aromatics are produced in He without CO_2_ per kilogram LDPE-1 (which contains 71.4 mol C), and 31.2 mol C of alkanes. In the presence of CO_2_, hydrogen is scavenged by CO_2_, which results in formation of 48.2 mol C of aromatics. Thus, the aromatics C yield increases by 23.0 mol by inhibiting alkane formation, including additional incorporation of 4.5 mol CO_2_ with respect to the reaction in He (Table S4). Thus, this new strategy for upcycling polyolefins and CO_2_ turns both waste materials into carbon resources for synthesis of value-added aromatics, particularly BTX. By using this approach, ∼0.63 kg aromatics, 0.15 kg liquefied petroleum gas (LPG) and 0.13 kg naphtha (Table S6) can be obtained, and 0.2 kg CO_2_ is consumed per 1.0 kg plastics based on the data in Fig. 1. By contrast, conventional catalytic cracking and hydrocracking processes without CO_2_ give only half that aromatics-yields.

## CONCLUSION

This study demonstrates for the first time that upcycling of polyolefin plastics can be combined with CO_2_ neutralization under mild conditions (below 300°C), enabled by the bifunctional catalyst of Pt/MnO_x_-ZSM-5. For example, 1.0 kg plastics and 0.2 kg CO_2_ are converted to 0.63 kg aromatics, 0.28 kg LPG and naphtha. The presence of CO_2_ not only inhibits alkane formation, but also drives the reaction towards aromatization by scavenging the hydrogen species generated from aromatization under the catalysis of Pt/MnO_x_. The aromatic-yield is as high as 64% and value-added BTX accounts for 60% among aromatics at 300°C, which is almost two times higher than the corresponding yields obtained in conventional catalytic cracking or hydrocracking over the same catalyst. Furthermore, CO_2_ is not only a hydrogen scavenger suppressing alkane formation but also a carbon source being incorporated into the aromatic products, which consequently boosts the aromatization of olefinic intermediates significantly. A higher aromatics-yield and fixation of more CO_2_ can be anticipated if a more efficient bifunctional catalyst is designed with a higher activity for CO_2_ hydrogenation.

## METHODS

### Catalytic reaction

Pt/MnO_x_ was reduced by H_2_ (10 vol% H_2_, 90 vol% Ar) in a tubular furnace before the reaction. Typically, the catalyst was heated in H_2_ at a ramp rate of 2°C/min to 320°C and maintained for 2 h. Then Pt/MnO_x_ was mixed with ZSM-5 upon grinding. In a typical reaction, 0.4 g Pt/MnO_x_-ZSM-5 composite catalyst was mixed with polyolefin powder (1.0 g) upon grinding in a mortar until a homogeneous color was achieved for the mixture. The mixture was loaded into a 100 mL batch reactor with an inner diameter of 35 mm, purchased from the Parr Instrument Company (Microreactor Model 4598). The reactor was heated by a heating jacket and equipped with a temperature programmed controlling system with a thermocouple placed inside the reactor. Note that under the reaction conditions, most products are in gas phase. Prior to reaction, the reactor was flushed three times by the feed CO_2_ with 95 vol% CO_2_ and 5 vol% Ar, with Ar as the internal standard for gas chromatography (GC, Agilent 7890B) analysis. The reaction was usually allowed to take place for 5 h. Subsequently, the reactor was cooled down to room temperature and the gaseous effluents were collected into a sampling bag. 15–30 mL dichloromethane (with 600 ppm n-Hendecane as the internal standard) was injected into the reactor to wash the reactor and the catalyst to dissolve any hydrocarbon products. The insoluble substance was collected by centrifugation and dried at 60°C. The resulting solid contains the catalyst, and carbon deposition and unreacted plastics, which were quantified by thermogravimetric analysis (TG).

Hayesep Q and 5 Å molecular sieve packed columns were connected to thermal conductivity detector (TCD) while HP-AL/S capillary columns were connected to FID-1, and an HP-FFAP capillary column connected to FID-2. C_1_−C_8_ hydrocarbons were analyzed by FID-1. Oxygen-containing compounds and hydrocarbons up to C_17_ were analyzed by FID-2, while CO, CO_2_, Ar, CH_4_, C_2_H_4_ and C_2_H_6_ were analyzed by TCD. C_2_H_4_ and C_2_H_6_ were taken as a reference bridge between FID and TCD.

CO_2_ conversion was calculated on a carbon atom basis:


(1) \begin{eqnarray*}
Con\left( {C{{O}_2}} \right) = \frac{{C{{O}_2}_{\textit{inlet}} - C{{O}_2}_{\textit{outlet}}}}{{C{{O}_2}_{\textit{inlet}}}} \times 100\%,
\end{eqnarray*}

where $C{{O}_2}_{\textit{inlet}}$ and $C{{O}_2}_{\textit{outlet}}$ represent moles of CO_2_ at the inlet and outlet, respectively.

The yield of hydrocarbon C*_n_H_m_* was calculated by adding the corresponding yield in both gaseous and liquid products:


(2) \begin{eqnarray*}
\textit{Yiel}{{d}_{{{\rm C}_n}{{\rm H}_m}}}\!\! =\! \textit{Yiel}{{d}_{( {{{\rm C}_n}{{\rm H}_{m\ }}\ in\ Gas} )}} \!+\! \textit{Yiel}{{d}_{( {{{\rm C}_n}{{\rm H}_{m\ }}{{in\, Liquid}}} )}}.
\end{eqnarray*}

The yield of *C_n_H_m_* in gas or liquid was calculated according to the ratio of the carbon mole number of *C_n_H_m_* to the total carbon mole number of converted PE and CO_2_ in the reaction, assuming complete conversion of PE.


(3) \begin{eqnarray*}
\textit{Yiel}{{d}_{{{\rm C}_n}{{\rm H}_{m\ }}\ }} = \frac{{n{{\rm C}_n}{{\rm H}_m}}}{{\ {{n}_{PE}} + {{n}_{\rm CO_{2 - \mathrm{conv}}}}}} \times 100\%.
\end{eqnarray*}

${{n}_{PE}}$ and ${{n}_{\rm CO_{2 - \mathrm{conv}}}}$ represent the converted PE and CO_2_, respectively. According to the measured residual solid after the reaction (deduced by the catalyst mass), the carbon balance was calculated to be >85% in this study.

## Supplementary Material

nwae097_Supplemental_File
