# Supplementary material for: CO2-facilitated upcycling of polyolefin plastics to aromatics at low temperature
Source: Natl Sci Rev. 2024 Mar 15;11(5):nwae097. doi: 10.1093/nsr/nwae097 (PMC11042496; doi:10.1093/nsr/nwae097)
Supplement: nwae097_Supplemental_File [file nwae097_supplemental_file.pdf]

Supplementary information

CO_2-facilitated upcycling of polyolefin plastics to aromatics at low temperature.

Yi Ding[1], Shuchi Zhang[1,2], Cheng Liu[1,2], Yu Shao[1,3], Xiulian Pan[*,1], Xinhe Bao[1,3]

[1]State Key Laboratory of Catalysis, Dalian Institute of Chemical Physics, Chinese Academy of Sciences, Zhongshan Road 457, Dalian 116023, China.
[2]University of Chinese Academy of Sciences, Beijing 100049, China.
[3]University of Science and Technology of China, JinZhai Road 96, Hefei 230026, China

*Email: panxl@dicp.ac.cn;

METHODS

MATERIALS

Polyethylene was denoted as PE. Low-density polyethylene with a relative molecular weight of $M_w = 2.9\times10^5$ g mol^{-1} (denoted as LDPE-1), high-density polyethylene with $M_w = 2.5\times10^5$ g mol^{-1} (denoted as HDPE), and polypropylene with $M_w = 4.7\times10^4$ g mol^{-1} (denoted as PP) in powders were purchased from Macklin. Low density polyethylene powder with $M_w = 4.8\times10^5$ g mol^{-1} (denoted as LDPE-2) was purchased from Alfa Aesar. A kitchen PE film with $M_w = 3.5\times10^5$ g mol^{-1}, which was used for wrapping of water beakers, was cleaned by water and ethanol, then dried and further cut into mm-size pieces before use. Polypropylene bottles with $M_w = 6.8\times10^6$ g mol^{-1}, which were laboratory sample containers, were cleaned by water and ethanol, then dried and further cut into mm-size pieces before use.

Aqueous solutions of H_2PtCl_6 (38wt% Pt) and $Mn(NO_3)_2$ (50 wt% Mn), and NH_3HCO_3 were purchased from Sinopharm Chemical Reagent. N-hendecane (≥99%) was purchased from Aladdin. Dichloromethane (HPLC, ≥99.5%) was purchased from Kermel.

Commercial ZSM-5 (SiO_2/Al_2O_3 ratio = 25) zeolites were purchased from Nankai University Catalyst Company, China.

CATALYSTS PREPARATION

MnO_x oxide was prepared by precipitation following the previously reported procedure[1]. 35.8 g $Mn(NO_3)_2$ aqueous solution (50 wt%) was diluted in 65 mL distilled water and 1.0 M NH_3HCO_3 aqueous solution was used as the precipitant. Precipitation was conducted at room temperature, followed by aging for 0.5 h at the same temperature. After filtering and washing by distilled water, the resulting sample was dried overnight at 110 ℃. Then it was calcined in static air, being heated at a ramp of 2 ℃/min to 400 ℃ and then maintained for 2 h.

Pt/MnO_x was prepared by wet impregnation using H_2PtCl_6 aqueous solution (20 mL 0.01 mol$_{(Pt)}$/L) and MnO_x oxide (4 g) with Pt loading being targeted at 1.0 wt%. The mixture was dried at 60 ℃ until all water evaporated. Then it was calcined in static air, being heated at a ramp of 2 ℃/min to 400 ℃ and then maintained for 2 h. ICP-OES measurement indicates an actual loading of 1.09 wt%, equivalent to a Pt/Mn molar ratio of 0.004 in the final Pt/MnO_x catalyst. Furthermore, Pt/MnO_x catalysts with Pt loading of 0.51 wt% and 0.21 wt% were also prepared for comparison by changing the concentration of H_2PtCl_6 aqueous solution.

MODEL REACTION

Model reaction of 1-hexene with CO_2 was carried out following the same procedure and the same conditions as the reaction of polyolefins with CO_2.

Reaction of LDPE-1 in the presence of CO_2 was also tested in a flow through reactor made of stainless-steel reactor furnished with a quartz lining with a diameter of 4 mm (Scheme S2). Typically, 0.4 mg LDPE-1 was mixed with 0.24 g ZSM-5 under grinding and

then shaped to pellets with a size of 40-60 mesh. The catalyst was packed into the reactor as a fixed bed, which was supported by a quartz-fiber bed below. It was heated from room temperature to 300 ℃ at a ramp rate of 5 ℃/min. CO_2 with 5% Ar as the internal standard for online gas chromatography (GC) analysis was fed through the mixture of polyolefins and catalyst (10 mL/min). Reaction conditions were 1.0 MPa and 300 ℃ unless otherwise stated.

CATALYST CHARATERIZATION

X-ray diffraction (XRD) was measured on a PANalytical X'pert PPR diffractometer equipped with CuKα radiation source (λ = 1.5418 Å), operated at 40 mA and 40 kV. XRD patterns were recorded in the range of 2θ = 5–50°.

Thermogravimetric analysis (TG) was performed on Netzsch STA 449 F3. The mass loss was recorded while the catalyst was heated from 40 to 900 ℃ at a ramp of 10 ℃/min in a flowing air (100 mL/min).

Nitrogen adsorption-desorption was carried out on a Quantachrome NOVA 4200e. Before analysis, all samples were pretreated at 300 ℃ for 6 h under vacuum. Isotherms were recorded at liquid nitrogen temperature of 77 K.

Temperature programmed desorption of ammonia (NH_3-TPD) was performed on a Micromeritics AutoChem 2910 equipped with a TCD. The catalyst was first pretreated in a flowing Ar at 550 ℃ for 1.5 h. After cooling down to 100 ℃ in a flowing Ar, the sample was exposed to 5% NH_3/He at 100 ℃. Then the sample was swept by He flow at 100 ℃ till a stable TCD signal baseline was obtained. Subsequently, the temperature was increased from 100 to 800 ℃ at a ramp of 10 ℃/min.

Pyridine Fourier transform infrared spectroscopy (FTIR), in situ H-D exchange FTIR and in situ diffuse reflection Fourier transform infrared spectroscopy (DRIFTS) of CO_2 adsorption were performed on a Bruker Tensor 27 with a MCT detector.

For a typical Pyridine FTIR experiments, catalyst powder was pressed into a wafer with a diameter of 14 mm with a thickness less than 0.5 mm. Prior to pyridine adsorption, the sample was degassed under vacuum ($<10^{-2}$ Pa) at 450 ℃ for 1.5 h. The background spectrum was recorded after the cell had been cooled down to 250 ℃. Subsequently, the sample was exposed to pyridine vapor for 5 min at room temperature, followed by evacuation to $<10^{-2}$ Pa for 30 min. FTIR spectra were then recorded by accumulating 64 scans at a resolution of 4 cm^{-1}.

Prior to the in situ H-D exchange FTIR experiments, the sample was pretreated in H_2 at 350 ℃ for 2 h. The background spectrum was recorded after the cell have cooled down to 200 ℃. Subsequently, the sample was exposed to D_2 at the same temperature. Then the Si-OD-Al band was monitored by accumulating 32 scans at a resolution of 4 cm^{-1}.

Prior to the in situ DRIFTS of CO_2 adsorption, all samples were pretreated in H_2 at 350 ℃ for 2 h. The background spectrum was recorded after the cell have cooled down to 250 ℃ in Ar. Subsequently, the sample was exposed to CO_2. DRIFTS were then recorded by accumulating 64 scans at a resolution of 4 cm^{-1}. After 20 min, the sample was exposed to Ar at 250 ℃ to remove the physically adsorbed CO_2, followed exposure to H_2 (5 mL/min). DRIFTS were by accumulating 64 scans at a resolution of 4 cm^{-1}.

The molecular weights and molecular weight distributions of the polyolefin were determined by gel permeation chromatography (GPC) with the PL-GPC220 equipped with a 40-position autosampler and a high-sensitivity refractive index detector at 150 ℃ using

1,2,4-trichlorobenzene as the solvent and calibrated with polystyrene standard.

GC-MS analysis was carried out using (Agilent 7890A-7000B) equipped with a PONA capillary column or FFAP column.

High resolution transmission electron microscopy (HRTEM) characterization was carried out on a JEOL JEM-F200 Transmission Electron Microscope, which was equipped with two EDS analyzers, operating at an accelerating voltage of 200 kV.

$$C_{20}H_{40} \rightarrow 1.25\, C_7H_8 + 3.75\ C_3H_8$$

$$\Delta_r H_m = 2.1\ kJ/mol \quad \Delta_r G_m = -102.4\ kJ/mol \tag{S1}$$

$$C_{20}H_{40} \rightarrow\ 2.86\, C_7H_8 + 8.57\, H_2$$

$$\Delta_r H_m = 473.1\ kJ/mol \quad \Delta_r G_m = 144.0\ kJ/mol \tag{S2}$$

$$C_{20}H_{40} + 3.33CO_2 \rightarrow 3.33\, C_7H_8 + 6.67\, H_2O$$

$$\Delta_r H_m = 196.4\ kJ/mol \quad \Delta_r G_m = -7.2\ kJ/mol \tag{S3}$$

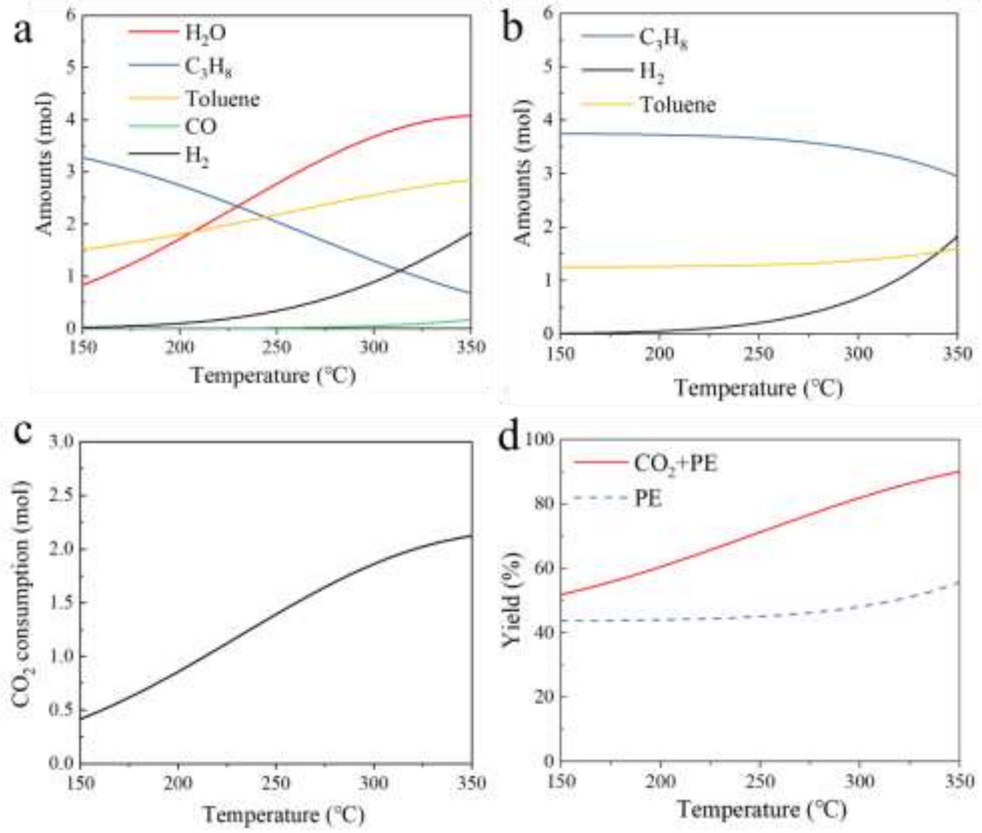

Figure S1. Thermodynamic calculation of C_{20}-Olefins conversion as a function of reaction temperature using HSC chemistry 9.0 software package. (a) Product distribution in the co-conversion of C_{20}-Olefins and CO_2; (b) Product distribution in the C_{20}-Olefins conversion without CO_2; (c) Thermodynamic equilibrium value of CO_2 consumption; (d) Comparison of aromatics yield in C_{20}-Olefins with CO_2 (red curve) and without CO_2 (blue curve) assuming C_3H_8, H_2, toluene C_7H_8 as products.

It can be seen from Figure S1c, thermodynamic equilibrium value of CO_2 consumption under 300 ℃ is 1.9 mol per 1 mol C_{20} olefin, the yield of aromatics can be increased from 48% to 82%, and the amounts of aromatics can be increased from 9.7 C mol to 18.2 C mol.

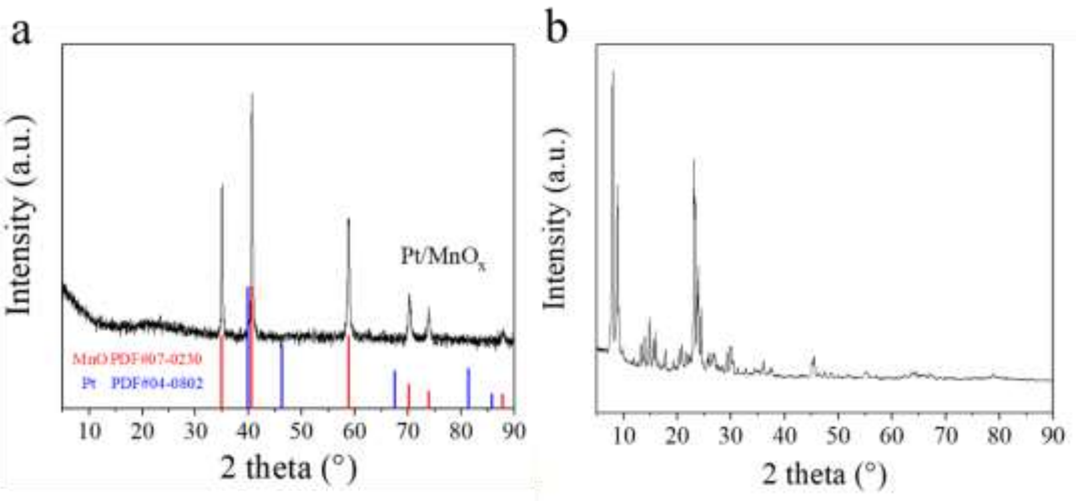

Figure S2. XRD patterns of catalysts. (a) Pt/MnO_x (pre-reduced); (b) ZSM-5.

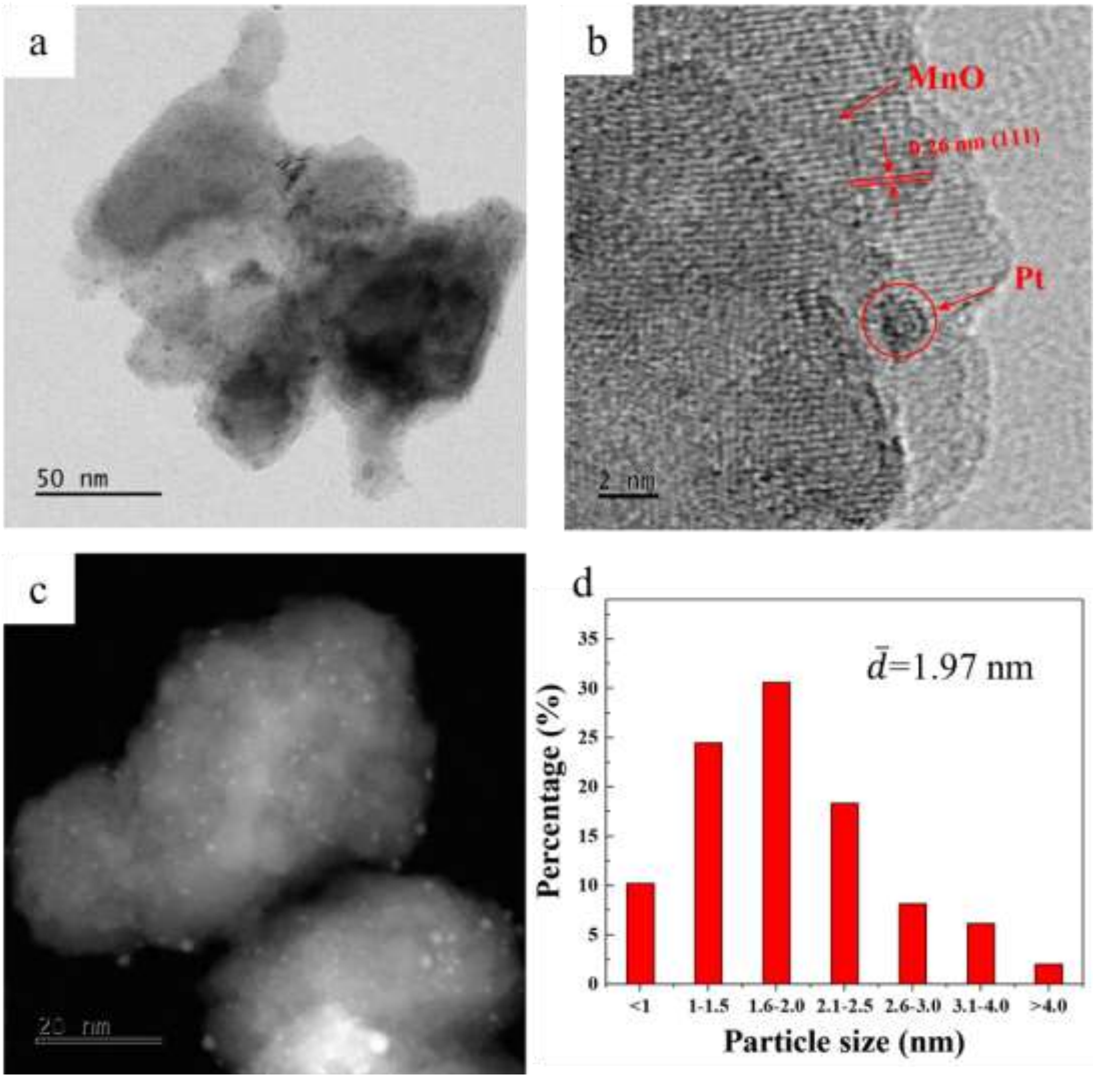

Figure S3. HRTEM images of fresh Pt/MnO_x (pre-reduced). (a) A typical image; (b) A high resolution image (c) High angle annular dark-field image; (d) Size distribution of Pt nanoparticles.

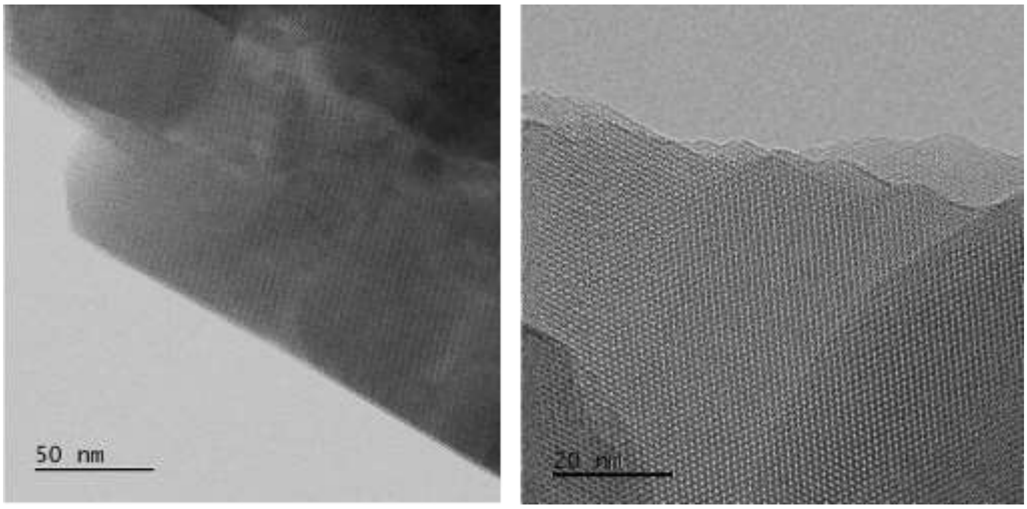

Figure S4. HRTEM of ZSM-5 zeolite.

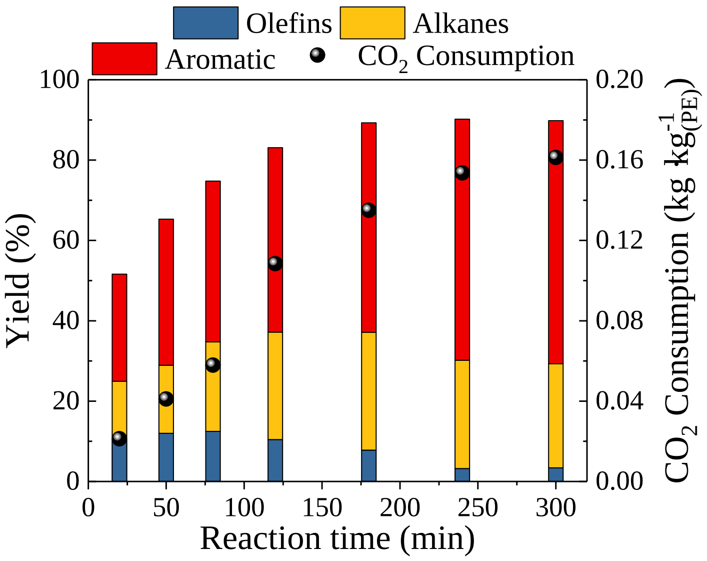

Figure S5. Catalytic performance of Pt/MnO_x-ZSM-5 as a function of reaction time at 280 ℃, 1.0 MPa CO_2, 0.4 g catalyst, 1.0 g LDPE-1, 100 mL batch reactor.

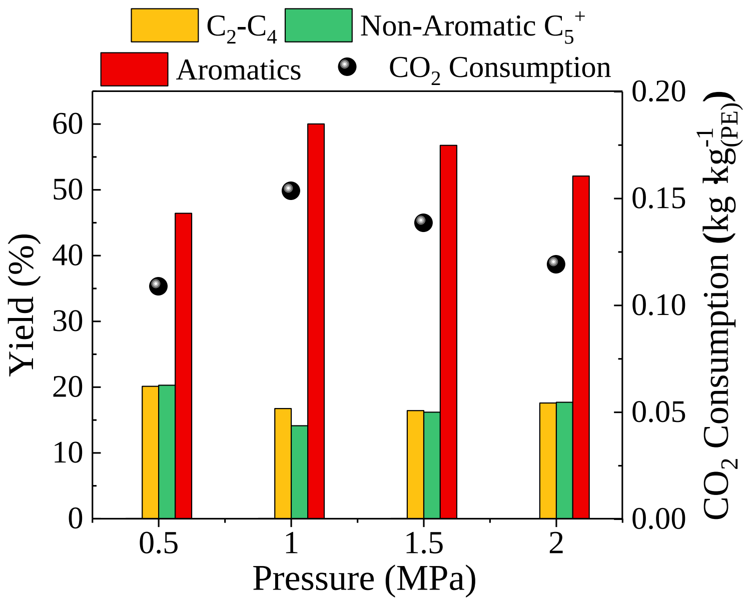

Figure S6. Catalytic performance of Pt/MnO_x-ZSM-5 in upcycling of LDPE-1 with CO_2 of different pressures. Reaction condition: 280 ℃, 0.4 g catalyst (Pt/MnO_x-ZSM-5), 1.0 g LDPE-1, 100 mL batch reactor.

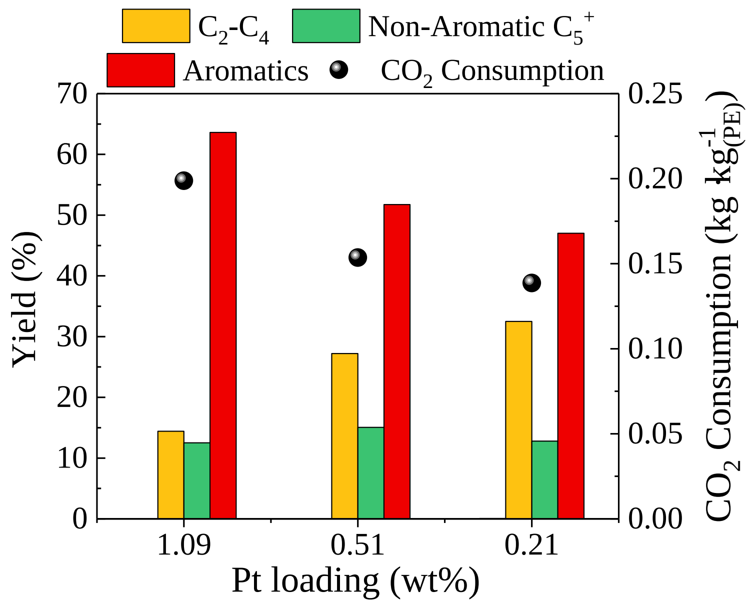

Figure S7. Catalytic performance of Pt/MnO_x-ZSM-5 in upcycling of LDPE-1 with CO_2 as a function of Pt loading on MnO_x. Reaction condition: 1.0 MPa CO_2, 300 ℃, 0.4 g catalyst (Pt/MnO_x-ZSM-5), 1.0 g LDPE-1, 100 mL batch reactor.

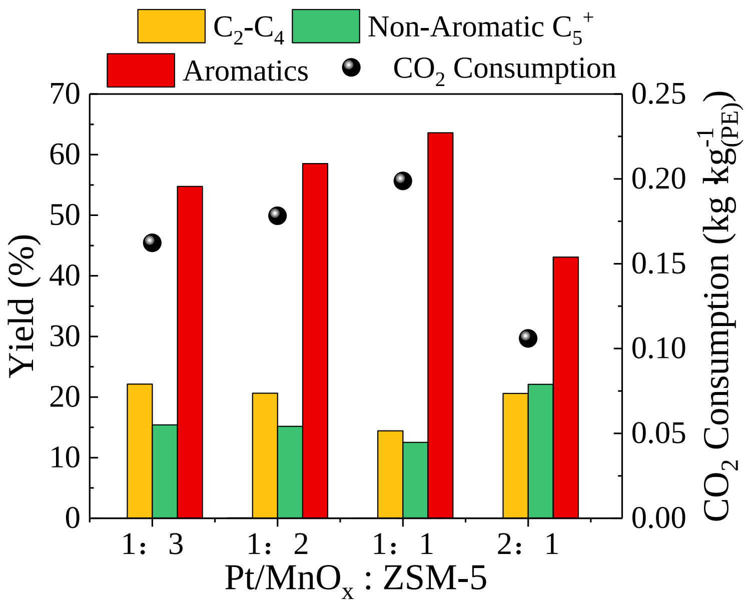

Figure S8. Catalytic performance of Pt/MnO_x-ZSM-5 in upcycling of LDPE-1 with CO_2 as a function of the mass ratio of Pt/MnO_x to ZSM-5. Reaction condition: 1.0 MPa CO_2, 300 ℃, 0.4 g catalyst (Pt/MnO_x-ZSM-5), 1.0 g LDPE-1, 100 mL batch reactor.

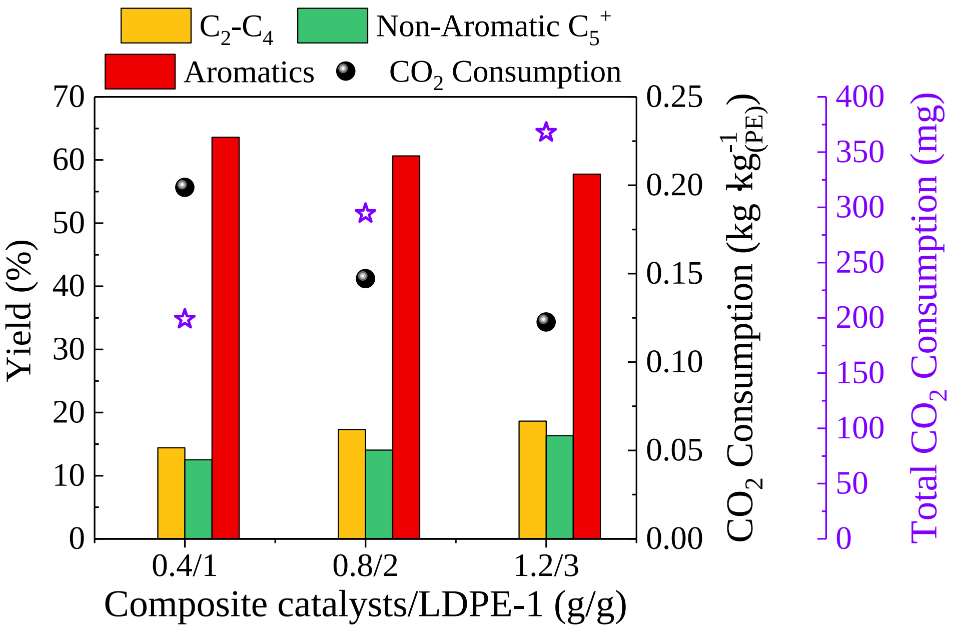

Figure S9. Reaction tests with different charges of composite catalyst/LDPE-1 (mass in gram) at 300 ℃ and 1.0 MPa CO_2 in 100 ml batch reactor. It shows that the total CO_2 consumption increases, the total yields of hydrocarbon products keep in the range of 91-93% and the yields of aromatics in the range of 57%-63%. The consumption of CO_2 per mass of LDPE-1 decreases and more alkanes form due to the lower CO_2/PE ratio.

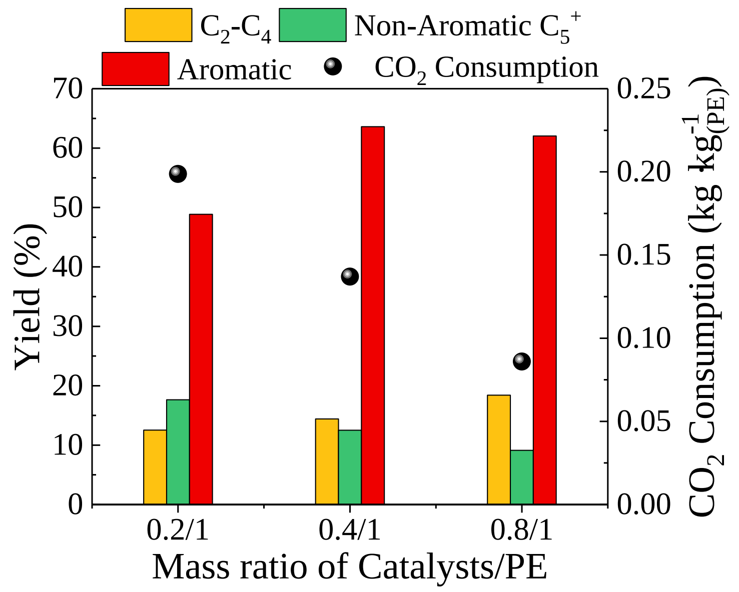

Figure S10. Reaction tests with different mass ratio of composite catalyst/LDPE-1, at 300 ℃ and 1.0 MPa CO_2 in 100 mL batch reactor.

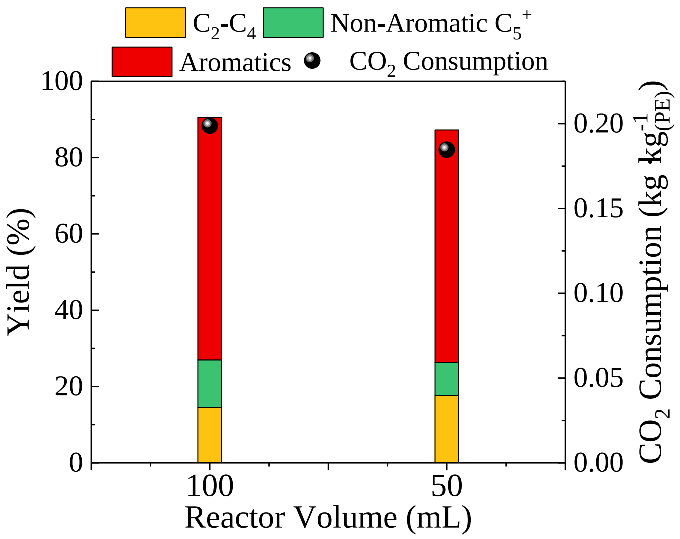

Figure S11. Reaction tests with a different volume of the batch reactor. Reaction conditions: 1.0 MPa CO_2, 300 ℃, 0.4 g catalyst/1.0 g LDPE-1 in 100 mL reactor and 2.0 MPa CO_2, 300 ℃, 0.4 g catalyst/1.0 g LDPE-1 in 50 mL reactor.

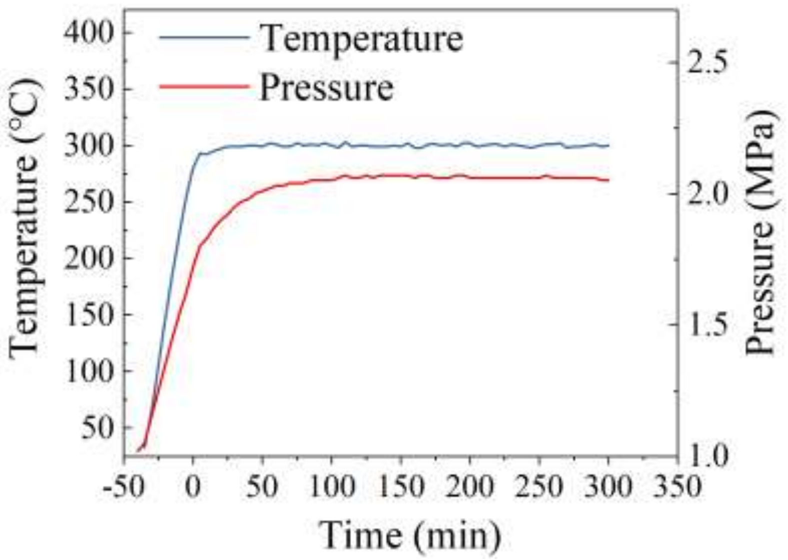

Figure S12 Evolution of pressure during upcycling of LDPE-1 with CO_2. Reaction conditions: 300 ℃ 1.0 MPa CO_2, 0.4 g catalyst (Pt/MnO_x-ZSM-5), 1.0 g polyolefins, 100 mL batch reactor.

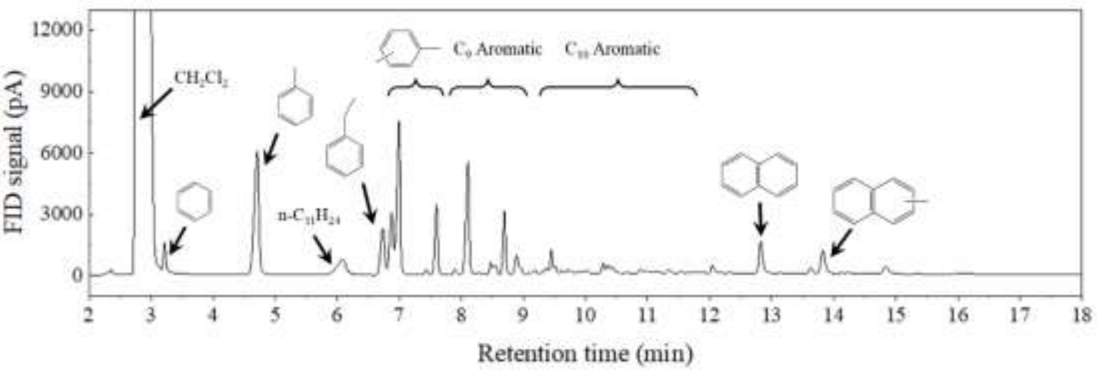

Figure S13. GC analysis of liquid products in the upcycling reaction of LDPE-1 with CO_2.

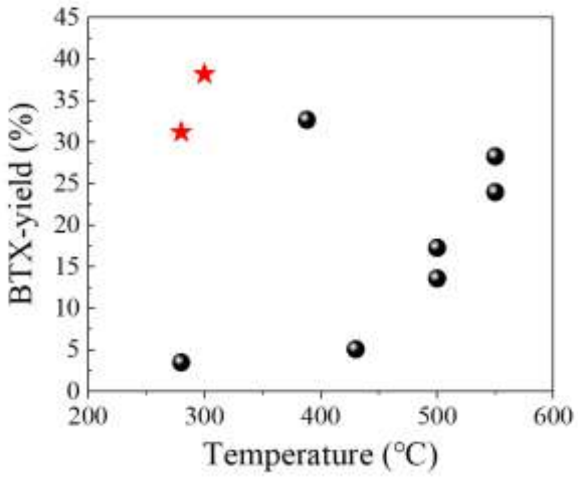

Figure S14. BTX-yield in this work (red stars) in comparison to the previously reported values (black circles) for one pot reactions versus reaction temperatures (corresponding to the data in Table S6).

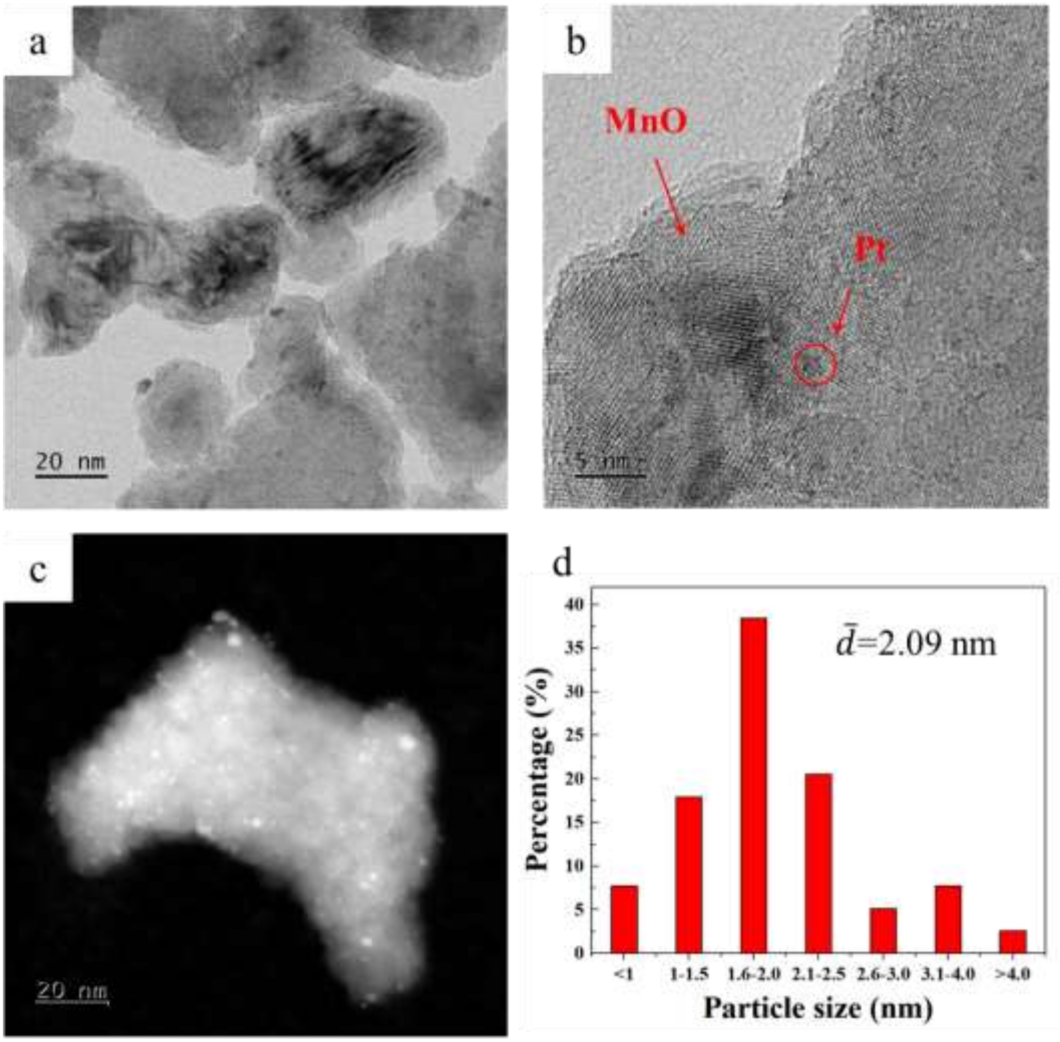

Figure S15. HRTEM of used Pt/MnO_x, which was separated from Pt/MnO_x-ZSM-5 after reaction for four cycles. (a) A typical TEM image; (b) A high resolution image; (c) High angle annular dark-field image; (d) Size distribution of Pt nanoparticles.

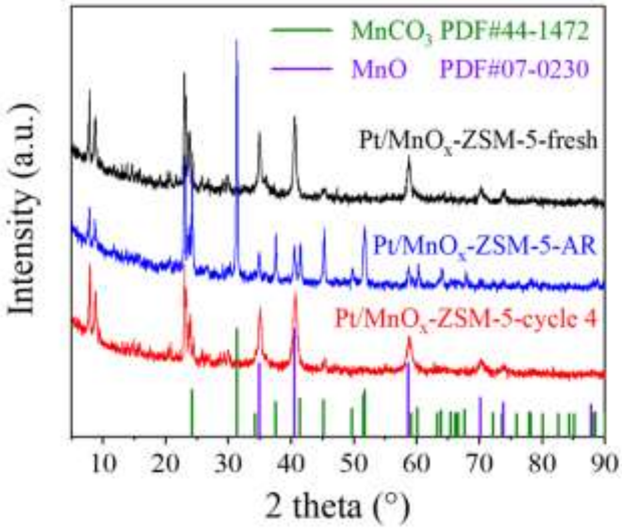

Figure S16. XRD patterns of Pt/MnO_x-ZSM-5 catalysts before and after reaction, and after regeneration. After reduction, only MnO crystal phase was observed. The reduced Pt/MnO_x was mixed with ZSM-5 as the fresh Pt/MnO_x-ZSM-5. After reaction, MnO was converted to $MnCO_3$, and the valence state of Mn remains +2, without further oxidation. In comparison, after 4 cycles of reaction and regeneration, no obvious crystallinity change was observed compared to the fresh Pt/MnO_x-ZSM-5 catalyst.

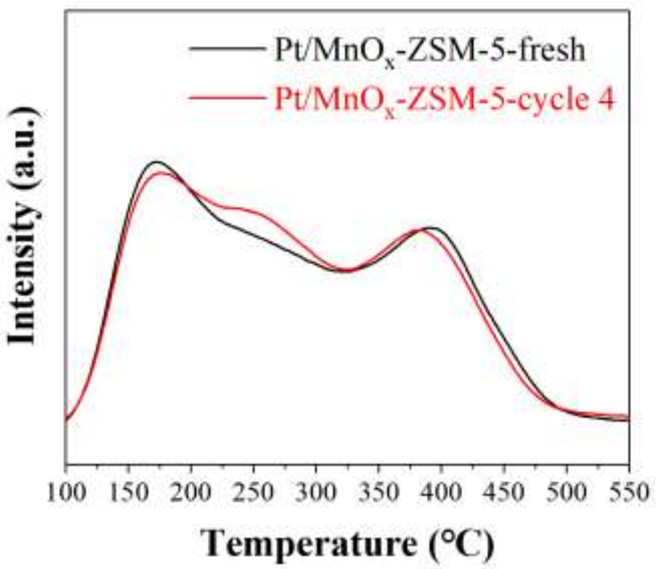

Figure S17. NH_3-TPD profiles of Pt/MnO_x-ZSM-5 catalysts before and after 4 cycles of reaction and regeneration.

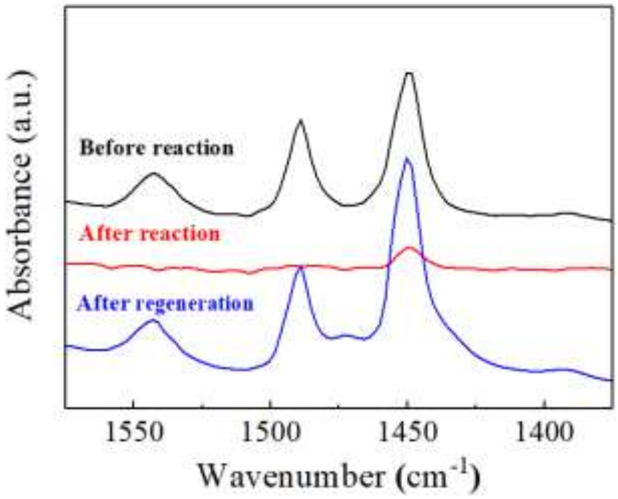

Figure S18. Pyridine adsorption of Pt/MnO_x-ZSM-5 catalysts before and after reaction and after regeneration.

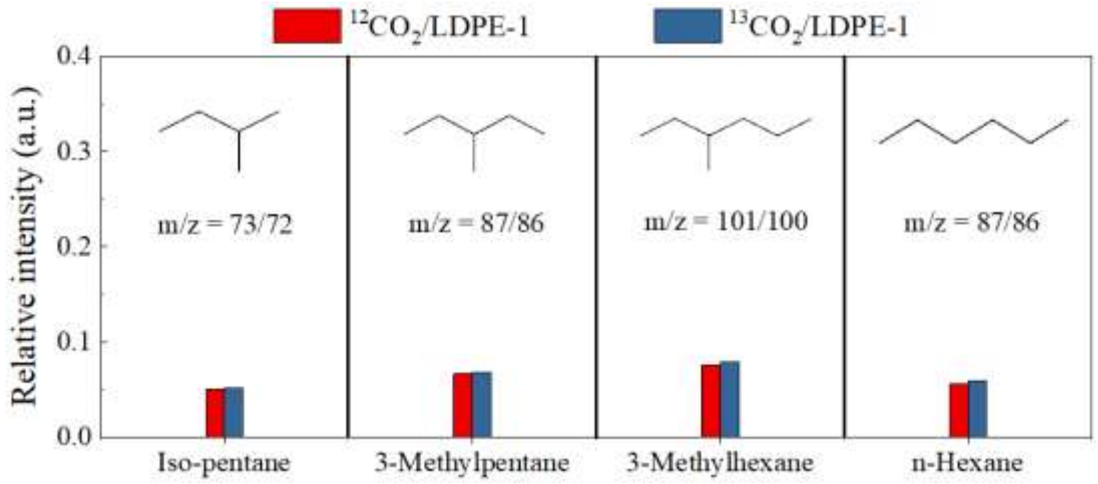

Figure S19. Mass spectra for detected alkanes during the reaction of LDPE-1 with $^{13}CO_2$ (blue) in comparison to that with $^{12}CO_2$ (red) at 300 ℃. (a) Isopentane; (b) 3-Methylpentane; (c) 3-Methylhexane; (d) n-Hexane.

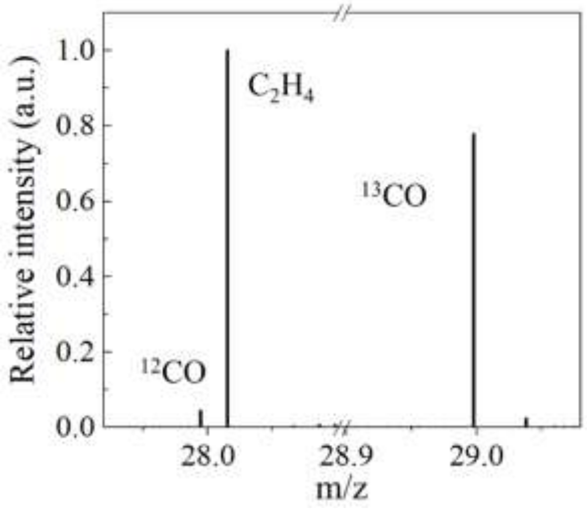

Figure S20. Mass spectra of ^{12}CO and ^{13}CO detected during the reaction of LDPE-1 with (a) $^{13}CO_2$ in comparison to that with (b) $^{12}CO_2$ at 300 ℃.

$$^{13}CO_2 + 2H^* \rightarrow {}^{13}CO + H_2O \quad \text{(S4)}$$

$$^{13}CO_2 + {}^{12}C^* \rightarrow {}^{13}CO + {}^{12}CO \quad \text{(S5)}$$

The detected CO in the reaction of LDPE-1 with $^{13}CO_2$ originates from two reactions, i.e. Equation S4 and S5. Therefore, one can estimate the amount of $^{13}CO_2$ turned into ^{13}CO according to the relative concentration of ^{13}CO among all CO measured by GC-MS analysis. It indicated that ^{13}CO accounted for 86% of all CO and the rest 14% CO was ^{12}CO, attributed to the reaction of $^{13}CO_2$ with carbon residual (Equation S5).

Thus, the fraction of CO_2 entering the aromatics ($S_{CO2\text{-}Aro}$) was calculated by Equation S6:

$$S_{CO2-Aro} = \frac{n_{CO2} - 0.86 * n_{CO}}{n_{CO2}} \quad \text{(S6)}$$

where n_{CO2} stands for the amount of converted CO_2 and n_{CO} for the total amount of CO, which were measured by GC. Thus, one can calculate that 90% of the consumed $^{13}CO_2$ had been incorporated into the aromatic products.

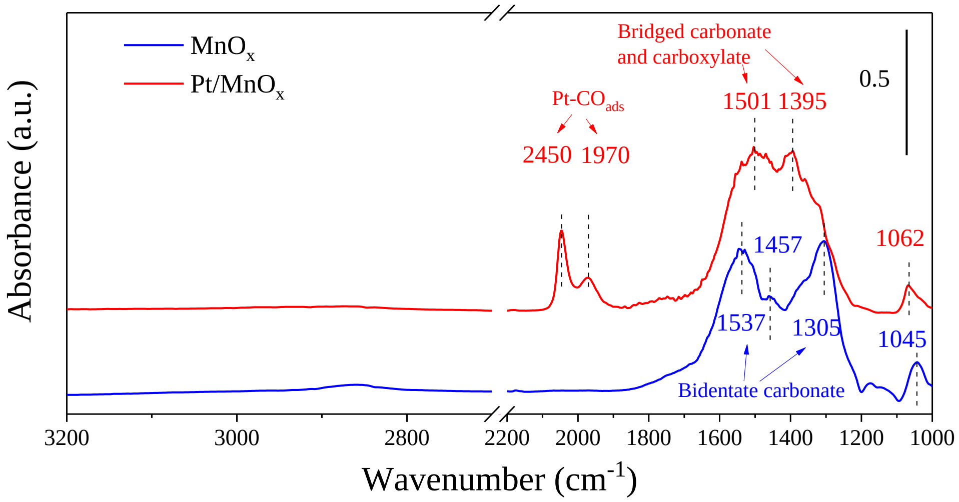

Figure S21. FT-IR differential spectra of CO_2 adsorbed Pt/MnO_x and MnO_x catalysts referenced to the catalysts prior to CO_2 adsorption. Stretching vibration of C-O at 1000 -2200 cm^{-1}; stretching vibration of C-H at 2800 -3200 cm^{-1}.

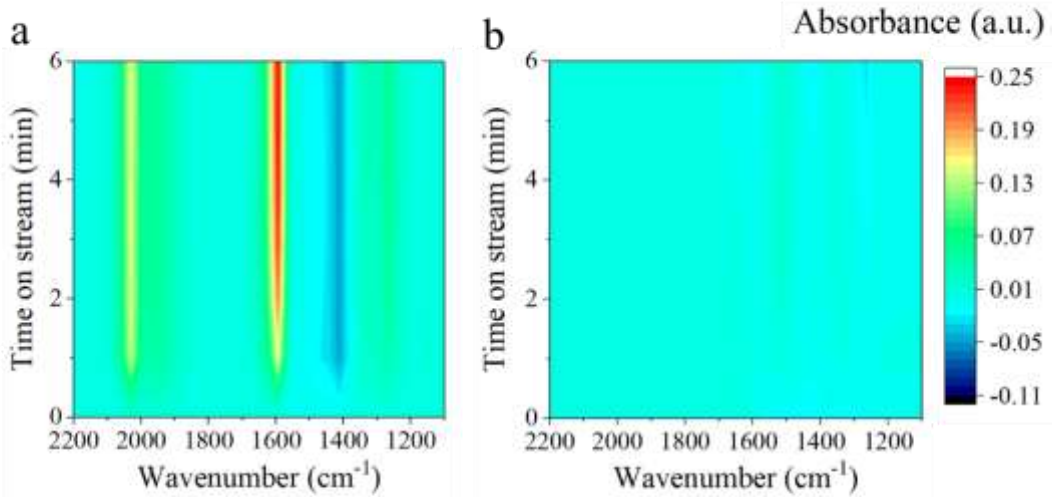

Figure S22. In situ IR differential spectra recorded after introduction of H_2 to the CO_2 adsorbed catalysts as a function of time on stream. (a) Pt/MnO_x (pre-reduced); (b) MnO_x (pre-reduced).

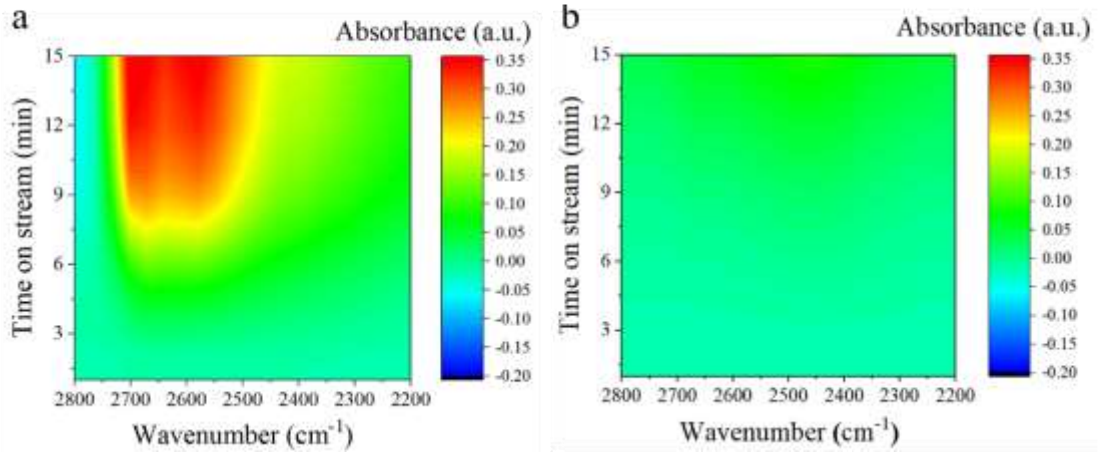

Figure S23. In-situ IR spectra showing Si-OD-Al signals during H-D exchange experiments. (a) Pt/MnO_x-ZSM-5; (b) ZSM-5.

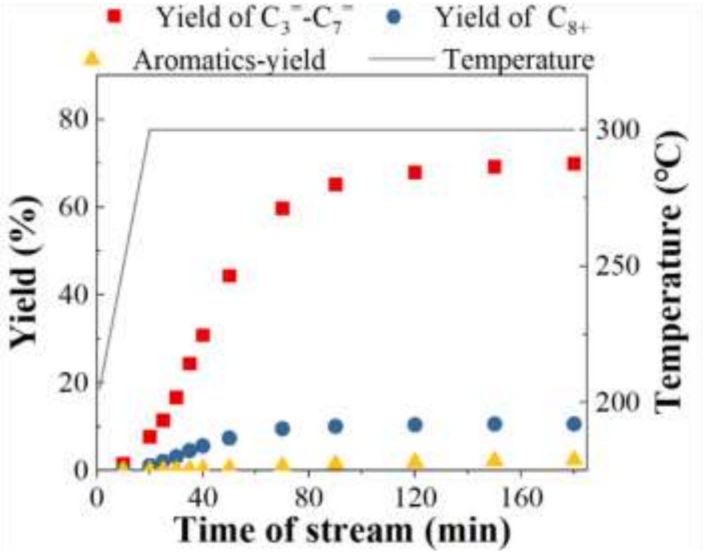

Figure S24. A fixed bed reaction with CO_2 flowing through the bed composed of the mixture of LDPE-1 and ZSM-5, with the effluents monitored by an online GC.

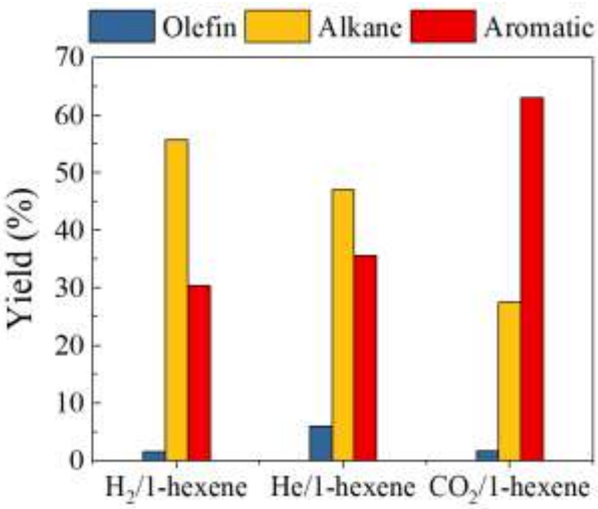

Figure S25. Control reactions of H_2/1-hexene and He/1-hexene in comparison to that of CO_2/1-hexene.

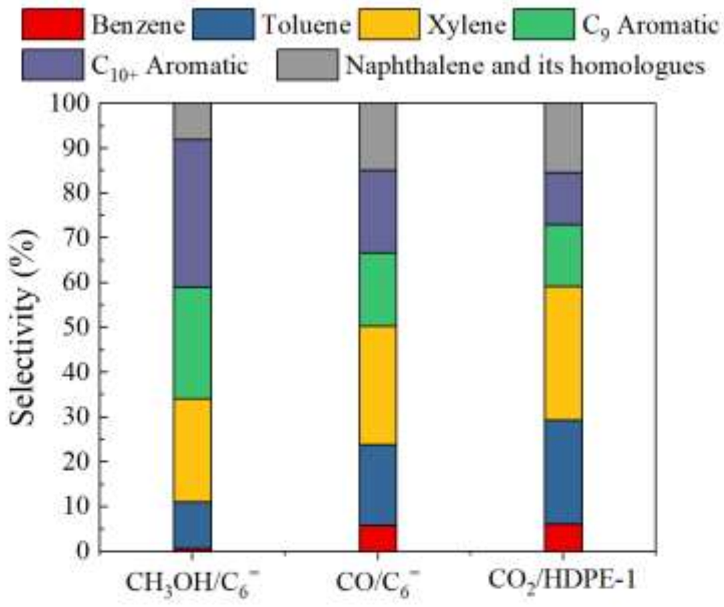

Figure S26. Detailed product distribution during the model reactions of $CH_3OH/C_6^=$ and $CO/C_6^=$ in comparison to CO_2/PE analyzed by GC.

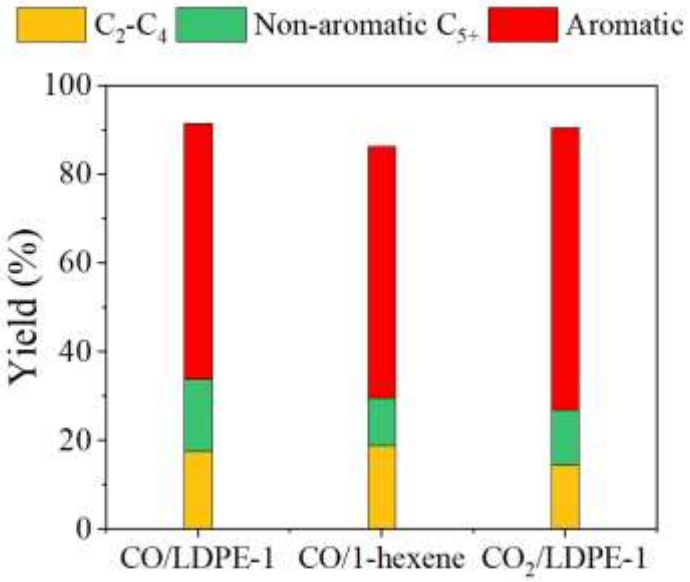

Figure S27. Reaction results of CO/LDPE-1 and CO/1-hexene in comparison to that of CO_2/LDPE-1. A similar product distribution further validates the proposed reaction mechanism in the paper.

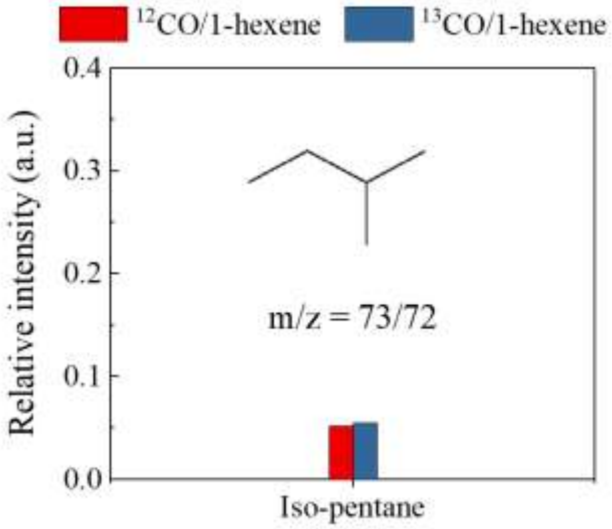

Figure S28. Mass spectra of alkane represented by isopentane during the reaction of ^{13}CO/1-hexene in comparison to that of ^{12}CO/1-hexene.

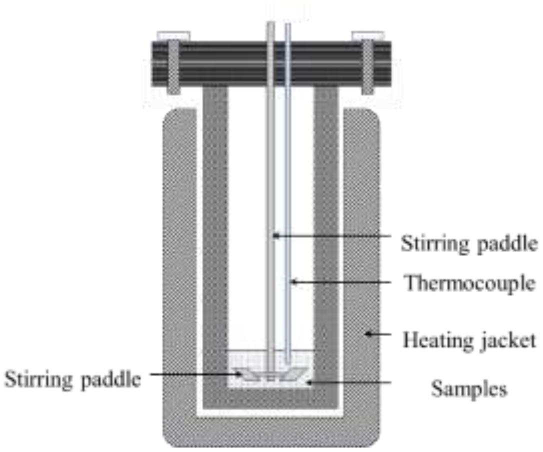

Scheme S1. Scheme for the batch reactor purchased from Parr Instrument Company (Microreactor Model 4598).

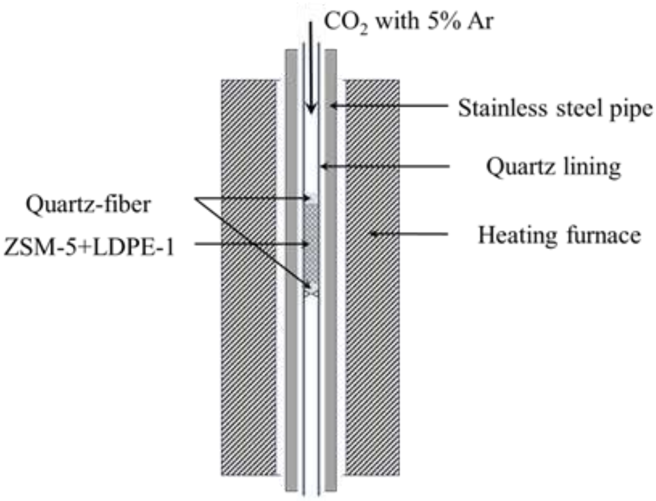

Scheme S2. Scheme of the flow-through reactor with the polyolefin-catalyst mixture packed as a fixed bed reactor and CO_2 flowing through the bed.

Table S1 Physical properties of ZSM-5.

**Sample**	**S_{BET} (m^2 g^{-1})**	**$S_{External}$ [b] (m^2 g^{-1})**	Pore Volume (cm^3 g^{-1}) [a]		**Density of Brønsted acid (mmol g^{-1}) [b]**	**Density of Lewis acid (mmol g^{-1}) [b]**
			V_{total}	V_{micro}		
**ZSM-5**	386.1	79.6	0.26	0.13	1.01	0.56

a: Calculated by t-plot method.

b: Determined by pyridine adsorbed FT-IR.

Table S2. Physical properties of commercial polyolefin samples

Samples	Source	M_w (g mol^{-1})	Particle size
**LDPE-1**	Macklin, China	2.9×10^5	~13 μm
**LDPE-2**	Alfa Aesar	4.8×10^5	≤ 400 μm
**HDPE**	Macklin, China	2.5×10^5	~ 74 μm
**PP**	Macklin, China	4.7×10^4	~ 74 μm
**PE film**	Hanshiliujia, China	3.5×10^5	mm-size
**PP bottles**	Gulei, China	6.8×10^6	mm-size

Table S3. Catalytic performance of different catalysts.[a]

Sample	CO_2 Consumption ($kg \cdot kg_{(PE)}^{-1}$)	Yield (%)				
		CO	Olefins	Alkanes	Aromatics	Solid[b]
Pt/MnO_x	--	--	5	3	1	80
ZSM-5	0.01	0.0	1	50	37	8
MnO_x-ZSM-5	0.05	0.2	2	40	44	6
Pt/MnO_x-ZSM-5	0.15	0.6	3	27	60	5

a: Reaction conditions: 1.0 MPa CO_2, 280 ℃, 0.4 g catalyst, 1.0 g LDPE-1, 100 mL batch reactor, unless otherwise stated. Note that there are still a small amount of undetected products.

b: The solid residual after reaction, including carbon deposition and unreacted polyolefin, was quantified by TG.

Table S4. Upcycling of LDPE-1 with CO_2 in comparison to the processes in H_2 and He.[a]

Atmosphere	CO_2 Consumption ($kg \cdot kg_{(PE)}^{-1}$)	Yield (%)/Yield ($mol \cdot kg_{(PE)}^{-1}$)					Hydrogen balance (%)
		CO	Olefins	Alkanes	Aromatics	Solid[b]	
**H_2**	--	--	1 (0.8)	51 (36.6)	35 (25.0)	5 (3.6)	--
**He**	--	--	4 (2.8)	44 (31.2)	35 (25.2)	7 (5.9)	86
**CO_2**	0.20 (4.5)	0.6 (0.5)	2 (1.3)	25 (18.5)	64 (48.2)	4 (3.2)	89

a: Reaction conditions: 1.0 MPa CO_2, 300 ℃, 0.4 g catalyst, 1.0 g LDPE-1, 100 ml batch reactor, unless otherwise stated. Note that there is a small amount of undetected products.

b: The solid residual after reaction, including carbon deposition and unreacted polyolefin, was quantified by TG.

Table S5. Aromatics-yield in comparison to those reported in literature for one pot reaction.

Catalysts	Feedstock	Temperature (℃)	Aromatics-yield (%)	BTX-yield (%)	References
**HZSM-5**	HDPE	430	5.1	5.1	[2]
**Pt/γ-Al2O3**	LDPE	280	46.0	--	[3]
**Ru/HZSM-5**	HDPE	280	44.5	3.5	[4]
**HZSM-5**	LDPE	550	32.3	24	[5]
**Al_2O_3+ZSM-5**	LDPE	550	45.6	28.3	[6]
**DeBeta**	HDPE	500	20.7	13.6	[7]
**DeBeta**	PP	500	25.0	17.3	[7]
**Zn(1)-ZSM-5**	LDPE	388	51.0	32.7	[8]
**This work**	LDPE	280	60.0	31.2	--
**This work**	LDPE	300	64	39.7	--

Table S6. Carbon balance during upcycling of LDPE-1 with CO_2 over Pt/MnO_x-ZSM-5.[a]

Reactant/Products	mol	g
**Converted CO_2**	4.5 mol	199 g
**-CH_2-(PE)**	71.4 mol	1000 g
**Products**		
**CO**	0.5 mol	13 g
**Coke[b]**	3.2 mol	38 g
**Aromatics**	5.9 mol (48.2 C mol)	626 g
**LPG**	3.1 mol (10.5 C mol)	147 g
**Naphtha**	1.7 mol (9.3 C mol)	131 g
**Undetected product[c]**	4.3 mol	--

a: Corresponding to the reaction at 300 ℃ in Figure 1b.

b: Assuming that all the solid residual collected after reaction is coke.

c: Obtained from the carbon balance.

References

1. Zhu Y, Pan X, Jiao F *et al.* Role of Manganese Oxide in Syngas Conversion to Light Olefins. *ACS Catal*. 2017; **7**: 2800-2804.
2. Garforth AA, Lin YH, Sharratt PN *et al.* Production of hydrocarbons by catalytic degradation of high density polyethylene in a laboratory fluidised-bed reactor. *Appl Catal A-Gen*. 1998; **169**: 331-342.
3. Zhang F, Zeng M, Yappert RD *et al.* Polyethylene upcycling to long-chain alkylaromatics by tandem hydrogenolysis/aromatization. *Science*. 2020; **370**: 437-441.
4. Du J, Zeng L, Yan T *et al.* Efficient solvent- and hydrogen-free upcycling of high-density polyethylene into separable cyclic hydrocarbons. *Nat Nanotechnol*. 2023.
5. Tian X, Zeng Z, Liu Z *et al.* Conversion of low-density polyethylene into monocyclic aromatic hydrocarbons by catalytic pyrolysis: Comparison of HZSM-5, Hβ, HY and MCM-41. *J Clean*. 2022; **358**: 131989.
6. Dai L, Zhou N, Li H *et al.* Catalytic fast pyrolysis of low density polyethylene into naphtha with high selectivity by dual-catalyst tandem catalysis. *Sci Total Environ*. 2021; **771**: 144995.
7. Lee HW, Park Y-K. Catalytic pyrolysis of polyethylene and polypropylene over desilicated Beta and Al-MSU-F. *Catalysts*. 2018; **8**: 501.
8. Wang Y, Cheng L, Gu J *et al.* Catalytic pyrolysis of polyethylene for the selective production of monocyclic aromatics over the zinc-Loaded ZSM-5 Catalyst. *ACS Omega*. 2022; **7**: 2752-2765.
